# Effect of GelMA Hydrogel Coatings on Corrosion Resistance and Biocompatibility of MAO-Coated Mg Alloys

**DOI:** 10.3390/ma13173834

**Published:** 2020-08-30

**Authors:** Wenxian Weng, Weiwei Wu, Xiaoming Yu, Mingyue Sun, Zhensheng Lin, Muhammad Ibrahim, Huazhe Yang

**Affiliations:** 1 School of Fundamental Sciences, China Medical University, Shenyang 110122, China; 18102489629@163.com (W.W.); wwwu.mail@foxmail.com (W.W.); smilesmy@foxmail.com (M.S.); 15260226552@163.com (Z.L.); 2 School of Material Science and Engineering, Shenyang Ligong University, Shenyang 110159, China; xmyu530@hotmail.com; 3 Institute of Metal Research, Chinese Academy of Sciences, Shenyang 110016, China; mibrahim16b@imr.ac.cn

**Keywords:** MAO, Mg alloys, GelMA, hydrogel

## Abstract

Micro-arc oxidation (MAO) treatment is a simple and effective technique to improve the corrosion resistance for magnesium alloys. However, the presence of micro-pores and cracks on the coatings provides paths for corrosive ions to penetrate into and react with the substrate, limiting the long-term corrosion resistance. In this paper, we designed a composite coating with which GelMA hydrogel coatings with varying thicknesses were prepared on the surface of MAO-coated magnesium alloys via a dip-coating method, aiming to improve the biocorrosion resistance and biocompatibility. The surface morphology, the chemical composition of GelMA hydrogels, and the crystallographic structure of magnesium alloys were characterized by scanning electron microscope (SEM), Fourier-transform infrared spectroscopy (FTIR), and X-ray diffraction (XRD), respectively. The corrosion resistance and biocompatibility of all samples were evaluated through electrochemical and biological experiments. The results demonstrated that the addition of GelMA hydrogel could effectively seal the pores and improve the corrosion resistance and biocompatibility of MAO-coated magnesium alloys, especially for the sample with one layer of GelMA hydrogel, showing high cell proliferation rate, and its current density (I_corr_) was two orders of magnitude lower than that of the MAO coating. Besides, the balance mechanism between corrosion and protection was proposed. As a result, the GelMA hydrogel coatings are beneficial to the application of MAO-coated magnesium alloys in bone tissue engineering and other fields.

## 1. Introduction

In recent years, magnesium (Mg) and its alloys are attracting immense attention in the clinical applications owing to their excellent biocompatibility and suitable biomechanical compatibility [1,2,3,4,5,6,7]. However, the poor corrosion resistance of magnesium alloys leads to a series of biological problems, such as excessive degradation speed, production of H_2_, and alkalization in the microenvironment surrounding the implant, which gives rise to the loss of biological function of implants [8]. Therefore, it is necessary to control the degradation rate of magnesium alloys for realizing long-term implantation. At present, surface modifications are considered to be an effective way to improve the corrosion resistance of magnesium alloys [9,10,11,12,13,14]. Among all of the various surface modifications, the MAO coatings have been widely employed on Mg alloys due to its metallurgical adhesion to the substrate, good wear resistance, moderate corrosion resistance, and high hardness [15,16,17,18,19]. For example, Zhao et al. [20] compared the properties of AZ91 alloy before and after the micro-arc oxidation (MAO) treatment and found that MAO treatment significantly improved the wear resistance and corrosion resistance, greatly improving the surface performance. However, MAO coatings have a larger number of pores and micro-cracks generated during the coating formation process, and the presence of pores and micro-cracks on the coatings provides paths, which facilitate the corrosive ions to penetrate into and react with the substrate, accelerating corrosion, and the coatings composed of inert materials are not conducive to cell adhesion and growth [21,22]. For instance, Fischerauer et al. [23] investigated the degradation rate of uncoated and MAO-coated ZX50 magnesium alloy implants in rat femurs and found that the degradation rate of uncoated magnesium alloy was higher than that of MAO-coated magnesium alloy in the first 4 weeks, but there was a reverse trend in the following 4 weeks. The result confirmed that the MAO coatings could ameliorate the corrosion resistance actually, but the corrosion resistance of MAO coatings was only reflected in the initial period of time because of the existence of the porous structure in MAO coatings.

Therefore, it is necessary to carry out post-processing, such as pore sealing, further for MAO-coated magnesium alloys to improve the corrosion performance and provide longer protection. Recently, composite coatings are extensively proposed to reduce the risk of the corrosion of magnesium alloys [24,25,26]. Li et al. [27] deposited Ta_2_O_5_ film on MAO coating by atomic layer deposition (ALD) technology, which effectively sealed the micropores and microcracks of the MAO coating. Moreover, the current density (I_corr_)of the composite coating decreased three orders of magnitude than that of the substrate and MAO coating, improving corrosion resistance. Zeng et al. [28] fabricated MAO/poly(L-lactic acid) (PLLA) organic composite coatings on Mg-1Li-1Ca alloys by dip-coating and freeze-drying method in order to improve the corrosion resistance and cell compatibility of Mg-1Li-1Ca alloys. Yu et al. [29] prepared MAO/chitosan (CS) organic composite coatings on the surface of magnesium alloy. It not only confirmed that the composite coating improved the corrosion resistance of the alloy but also showed a noticeable inhibition on the growth of bacteria. Thereby, it is of importance to improve both the corrosion resistance of magnesium alloys and biocompatibility by means of forming the composite coatings prepared on MAO-coated magnesium alloys. As a hydrophilic three-dimensional polymer mesh material, GelMA hydrogels have attracted particular attention in tissue engineering owning to their stable physicochemical properties, good biocompatibility, and cell enzymatic degradation [30,31,32,33]. Tan et al. [34] applied GelMA/HA composite as a coating on titanium-based materials, and the results demonstrated that the coating had good bone repairability. Thereby, it has great potential in improving the corrosion resistance and biocompatibility theoretically if the GelMA hydrogels are used to seal the pores of the MAO coatings. Nevertheless, to the best of our knowledge, there is no report related to the composite surface modification of MAO-coated magnesium alloys with GelMA hydrogel coatings.

Herein, we fabricated a different thickness of GelMA hydrogel coating on the surface of MAO-coated WE43 alloys by the dip-coating method and obtained insight into the corrosion resistance and biocompatibility of MAO-coated magnesium alloys with and without GelMA hydrogel coatings.

## 2. Materials and Methods

### 2.1. Samples and Coating Preparation Procedures

**Figure 1:**
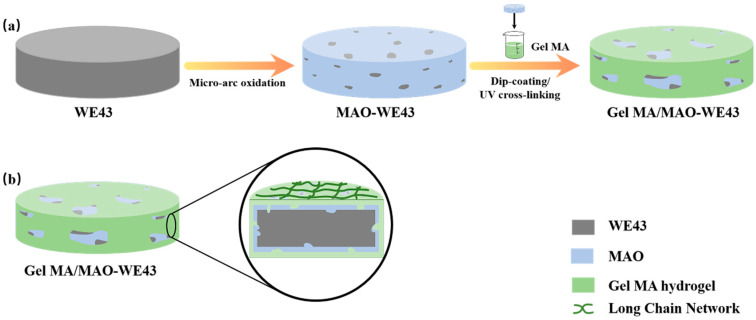
Schematic diagram illustrating the preparation process of the GelMA/MAO (micro-arc oxidation) composite coating on the WE43 magnesium alloy substrate: (**a**) WE43 magnesium alloy substrate pretreated by polishing, cleaning, and drying; the MAO coating prepared on the magnesium alloy substrate; the GelMA hydrogel coating prepared on the MAO-WE43 alloy with the dip-coating method; the GelMA/MAO-WE43 alloy pretreated by UV irradiation; (**b**) Section diagram of GelMA/MAO-WE43 alloy.

WE43 Mg alloys ingot (Mg-4% Y-3.3% RE (Nd, Gd)-0.5Zr%) were purchased from Wuxi Taicheng Metal Material Products Co. Ltd., and these materials were cut into pellets with Φ 10 mm × 1 mm for electrochemical tests, atomic force microscope (AFM, the AFM mode of a scanning tunneling microscope, CSPM5500, Benyuan nano Instrument Co. Ltd., Guangdong, China), scanning electron microscope (SEM, Zeiss, Germany), and Φ 8 mm × 1 mm for extract preparation, cell culture tests, and other assays. The preparation process of GelMA/MAO composite coating was carried out as follows, with a schematic diagram illustrated in Figure 1. Prior to MAO coating, the samples were polished with abrasive papers and washed with alcohol ultrasonically. The MAO process was carried out under the environment of 1400 Hz alternating current, 450 V voltage, and a duty cycle of 30% oxidized. The whole processing of MAO lasted 9 min at room temperature.

The GelMA hydrogel coating was prepared via a dip-coating method. Firstly, GelMA prepolymer (the synthesized process was described in Ref [35]) was dissolved in PBS at 37 °C for a 10 wt.% solution. The MAO-treated samples were ultrasonically cleaned in alcohol for 10 min and dried at room temperature. Subsequently, MAO-coated samples were dipped into the GelMA prepolymer solution for 60 s, withdrawn at a speed of 1 cm/min, and cross-linked in UV for 5 min to form GelMA hydrogel coating. The dipping repeated was set to 0, 1, 5, 10, and 15 times to obtain GelMA hydrogel coatings with different thicknesses. The prepared samples were kept drying in the air for at least 48 h for further assays. The prepared coated samples were as follows:

MAO-WE43, GelMA/MAO-WE43 with GelMA coating time of 1, 5, 10, 15. Therein, GelMA/MAO-WE43 with GelMA coating time of 1, 5, 10, 15 was named as 1GelMA/MAO-WE43, 5GelMA/MAO-WE43, 10GelMA/MAO-WE43, and 15GelMA/MAO-WE43, respectively. Single MAO-coated samples were used as control. The processing of samples is shown in Table 1.

### 2.2. Characterization of Coatings and Electrochemical Test

#### 2.2.1. Surface Analysis

The crystallographic structures of WE43 and MAO-WE43 samples were investigated by an X-ray diffraction meter (XRD, Cu Kα, D/max-A, Rigaku, Japan) at a scanning speed of 2°/min in the 2θ range of 20°–90°. The phase composition of GelMA hydrogel was analyzed by Fourier-transform infrared spectroscopy (FTIR-850, Tianjin Gangdong Technology Co. Ltd, Tianjin, China). The range of the spectra collected was 4000–400 cm^−1^. Then, a scanning electron microscope (SEM) was used to observe the morphology and layer thickness of the coated samples. In addition, the surface roughness of samples was analyzed through atomic force microscopy (AFM). The surface roughness (Ra) was estimated by using the following equation:

(1) $$\mathrm{Ra}=\frac{\int_{0}^{L}\left|r\left(x\right)\right|dx}{L}$$

where r(x) is a profile deviation from its mean value, and *L* is a sampling length.

#### 2.2.2. Electrochemical Corrosion Test

The electrochemical test was carried out on a multifunctional electrochemical workstation (Reference3000, Gamry, Philadelphia, PA, USA) with a three-electrode system that includes the coated samples as the working electrode (the exposed surface area was 0.785 cm^2^), a platinum plate as the counter electrode, and a saturated calomel electrode (SCE) as the reference electrode. The scanning rate was 1 mV·s^−1^ in the potentiodynamic polarization. The open circuit potential (OCP) was from −0.5 V vs. OCP to 0.5 V vs. OCP.

### 2.3. The Evaluation of Cytocompatibility In Vitro

#### 2.3.1. Preparation of Extracts

To gain the extracts, the coated samples were sterilized by ultraviolet irradiation at least 1 h and then immersed in cell culture solution (90% α-MEM (Hyclone, Logan, UT, USA) + 10% fetal bovine serum (Gibco, New York, NY, USA)) with 0.1% penicillin-streptomycin solution (and μg/mL, respectively, Hyclone). The immersion ratio of sample surface area to solution volume was 3 mL/cm^2^. Then, samples were all kept in the CO_2_ incubator (37 °C, 5% CO_2_) for 3 days. The Mg^2+^ content and pH value of extracts were analyzed by an Ultraviolet spectrophotometer (T6 new centroy, Beijing Puxi General Instrument Co. Ltd, Beijing, China) and a pH meter (FE20, Mettler Toledo, Göttingen, Germany), respectively. The extracts of samples were saved at 4 °C for biocompatibility tests.

#### 2.3.2. Cell Culture

MC3T3-E1 (Shanghai Zhong Qiao Xin Zhou Biotechnology Co., Ltd, Shanghai, China) were cultured in cell culture solution (the component of culture solution was like above) under the environment of CO_2_ incubator (37 °C, 5% CO_2_). When 80–90% of the bottom of the culture bottle was full of cells, the cells were detached by 0.25% trypsin-EDTA (Gibco) and subcultured until the third passage, which was used to the evaluation of cytocompatibility in vitro.

#### 2.3.3. Cell Viability and Proliferation Assay

In this experiment, MC3T3-E1 cells were firstly seeded in a 96-well plate for 24 h. The density and volume of cell suspension were 2 × 10^4^ cells/mL and 100 μL/well, respectively. After 24 h of incubation, the cell culture solution was replaced by the sample extracts. Then, the cell was continually incubated for 1, 3, and 5 days. When the experiment finished, 10 μL of CCK8 solution was added in each well and then incubated for 2 h in the CO_2_ incubator. Lastly, the optical density of each well was measured by a microplate reader (Model 680, Bio-Rad, California, America) at 450 nm.

## 3. Results

### 3.1. XRD Results and FTIR Spectra

**Figure 2:**
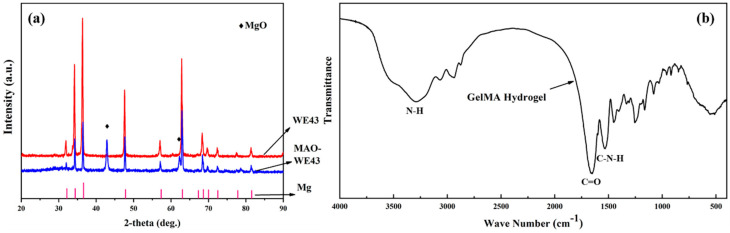
(**a**) XRD of WE43 and MAO-WE43, (**b**) FTIR of GelMA hydrogel.

Figure 2a illustrates the XRD patterns of WE43, MAO-WE43 samples. Obviously, compared with WE43 alloys, there was a MgO diffraction peak in MAO-WE43 samples, which was the result of plasma chemical oxidation reactions in the discharge channels produced by spark [16]. The diffraction angle corresponding to the diffraction peak of Mg was smaller than that of the standard card, which could be ascribed that there were other elements in WE43 besides Mg, but they were not detected due to their small content.

Figure 2b shows the FTIR patterns of GelMA hydrogel. FTIR designated that the peak at 3289 cm^−1^ indicated the presence of peptide bonds (mainly N–H stretching). The strong peak at 1653 cm^−1^ was related to C=O stretching vibration, and the peak at 1535 cm^−1^ was related to C–N stretching plus N–H bending. It was found that the GelMA hydrogels contained abundant amino and amide groups from the FTIR spectra, which also confirmed their high hydrophilicity.

### 3.2. The Morphologies of the Coatings

**Figure 3:**
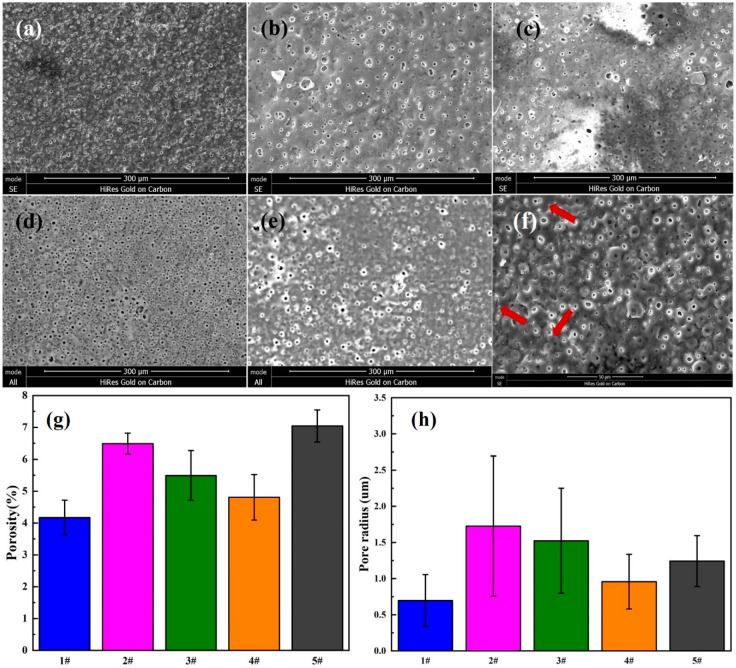
SEM images of the surface morphology (**a**–**e**), the high magnification (**f**), surface porosity (**g**) and pore size (**h**) of MAO-WE43 and GelMA/MAO-WE43 coatings. (**a**): 1#, MAO-WE43; (**b**): 2#, 1GelMA/MAO-WE43; (**c**): 3#, 5GelMA/MAO-WE43; (**d**): 4#, 10GelMA/MAO-WE43; (**e**): 5#, 15GelMA/MAO-WE43.

Figure 3 shows a comparison of SEM images of MAO-WE43 samples and four groups of GelMA/MAO-WE43 samples with varying layers of GelMA hydrogel coating (1,5,10,15) (a–e), also, the surface porosity (Figure 3g) and pore size (Figure 3h) of the five groups of samples. It could be seen that there were micropores with a size of about 0.7 μm and microcracks (Figure 3f) on the surface of MAO-WE43 coatings, resulting from discharge tunnels between electrolyte and surface during MAO process. As for samples added to GelMA hydrogel coatings, no obvious distinct was observed between MAO-WE43 samples and GelMA/MAO-WE43 samples with different layers in terms of porosity. The average hole radius of 1GelMA/MAO-WE43, 5GelMA/MAO-WE43, 10GelMA/MAO-WE43, and 15GelMA/MAO-WE43 samples was about 1.725, 1.5, 0.85, and 1.225 μm, respectively, which was larger than that of MAO-WE43 samples. Furthermore, the SEM images revealed that owing to the porous structure of GelMA hydrogels, all surface morphology of the composite coatings showed a poor coating uniformity with small island protuberances and asymmetrical pores, compared with MAO-WE43 samples. In general, increasing the number of the coating layers decreased the pore size and the pore size distribution; however, 15GelMA/MAO-WE43 samples had a bit larger pore size than the previous grade samples. According to the previous research theory [14,36] that the achieved layer thicknesses and the pore formation were influenced by the viscosity of the GelMA hydrogel or stress on the coated layer during extraction, we could speculate that uniformity and individual anomalies were the results of GelMA hydrogel viscosity and stress.

**Figure 4:**
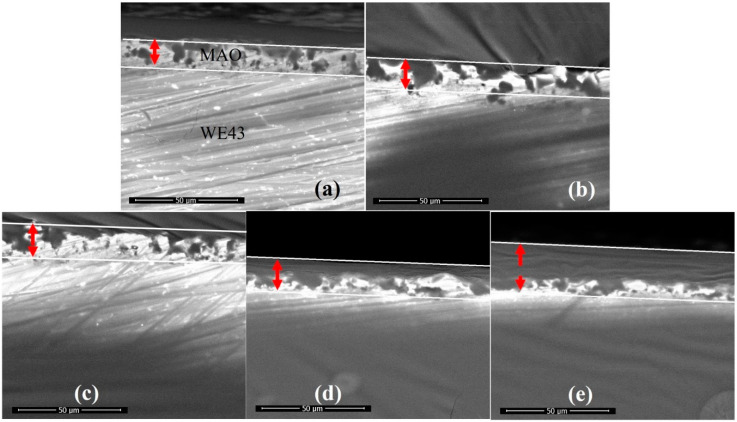
SEM cross-sectional morphology of MAO-WE43 and GelMA/MAO-WE43 coatings. (**a**): 1#, MAO-WE43; (**b**): 2#, 1GelMA/MAO-WE43; (**c**): 3#, 5GelMA/MAO-WE43; (**d**): 4#, 10GelMA/MAO-WE43; (**e**): 5#, 15GelMA/MAO-WE43.

**Figure 5:**
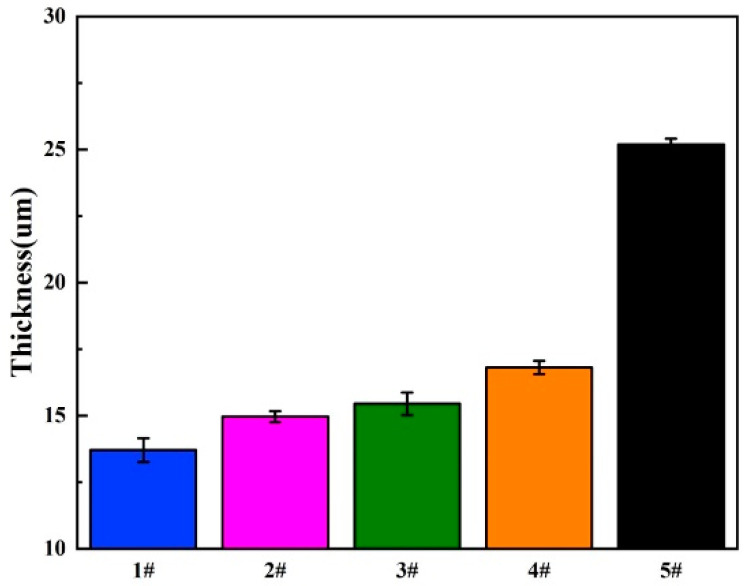
The coating thickness of MAO-WE43 and GelMA/MAO-WE43 samples. 1#: MAO-WE43; 2#: 1GelMA/MAO-WE43; 3#: 5GelMA/MAO-WE43; 4#: 10GelMA/MAO-WE43; 5#: 15GelMA/MAO-WE43.

As shown in Figure 4, the cross-section of GelMA/MAO-WE43 coatings presented the profile of the hierarchical structure. From the cross-section images, we can observe the distinct boundary between the GelMA layer and the MAO layer and the fuzzy connection between the WE43 magnesium alloy and the MAO layer. The thicknesses of samples were 13.7, 14.9, 15.4, 16.8, and 25.2 μm with the numbers of dip-coating of 0, 1, 5, 10, and 15 times on average, respectively (Figure 5, Table 2). As expected, the coating thickness increased with an increase in the number of dip-coating. In particular, the thickness of the 15GelMA/MAO-WE43 sample increased exponentially compared with that of 10GelMA/MAO-WE43. Therefore, it was reasonable to believe that the coating of GelMA hydrogel could effectively seal the pores and microcracks of MAO coatings and achieve the purpose of improving the corrosion resistance of materials.

**Figure 6:**
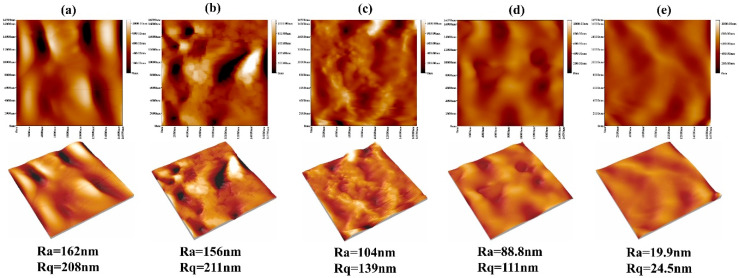
AFM images of MAO-WE43 and GelMA/MAO-WE43 samples. Ra: mean value of roughness; Rq: root mean square of roughness; (**a**): 1#, MAO-WE43; (**b**): 2#, 1GelMA/MAO-WE43; (**c**): 3#, 5GelMA/MAO-WE43; (**d**): 4#, 10GelMA/MAO-WE43; (**e**): 5#, 15GelMA/MAO-WE43.

**Figure 7:**
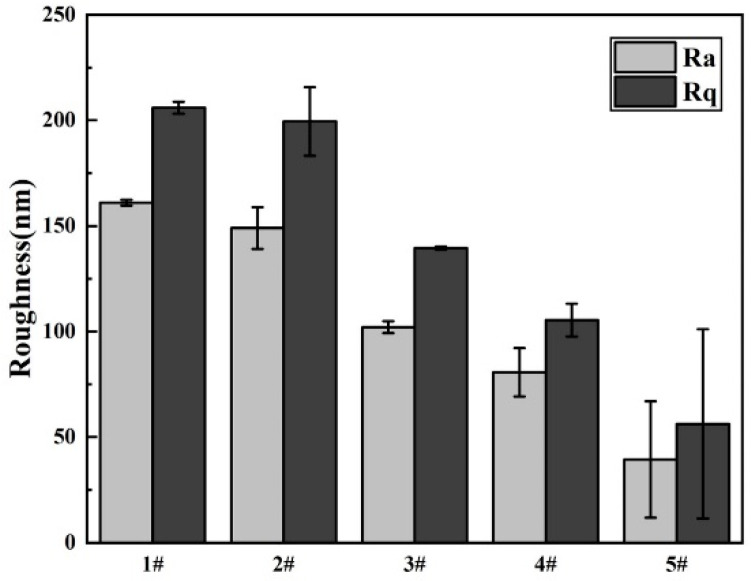
Surface roughness of MAO-WE43 and GelMA/MAO-WE43 samples. 1#: MAO-WE43; 2#: 1GelMA/MAO-WE43; 3#: 5GelMA/MAO-WE43; 4#: 10GelMA/MAO-WE43; 5#: 15GelMA/MAO-WE4.

As the surface roughness of implant materials exerted a pronounced influence on cell attachment, we investigated this property using atomic force microscopy (AFM), as depicted in Figure 6. The statistical analysis diagram of the surface roughness of samples is shown in Figure 7. From the three-dimensional and two-dimensional surface topography, it can be recognized that with the increase of the coating thickness, the surface roughness decreased correspondingly, which was closely related to the hydrophilicity of hydrogels. In addition, it was noted that the surface roughness of MAO-WE43 samples was higher than that of GelMA/MAO-WE43 samples, especially for 15GelMA/MAO-WE43 samples, showing a smooth surface. Therefore, the preparation of GelMA hydrogel coating with different thicknesses on MAO coating could change the surface water absorption degree and change the surface roughness of the composite coating then. However, it could not be ignored that the number and size of the pores on the coating surface could also affect the surface roughness.

### 3.3. Electrochemical Corrosion

**Figure 8:**
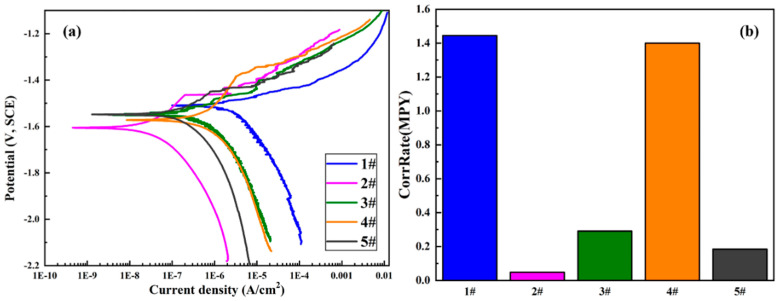
(**a**) PDP (potentiodynamic polarization) curves and (**b**)corrosion rate of MAO-WE43 and GelMA/MAO-WE43 samples. 1#: MAO-WE43; 2#: 1GelMA/MAO-WE43; 3#: 5GelMA/MAO-WE43; 4#: 10GelMA/MAO-WE43; 5#: 15GelMA/MAO-WE43.

Electrochemical methods are extensively used in studying corrosion behaviors as they are suitable for evaluating the corrosion mechanisms of the samples. Herein, potentiodynamic polarization (PDP) was carried out to probe the effect of GelMA hydrogel coatings on the corrosion behaviors of the MAO-coated magnesium alloys. The PDP curves are displayed in Figure 8, and relevant electrochemical parameters are summarized in Table 3. It can be seen that the I_corr_ of the samples followed this consequence: 1GelMA + MAO < 15GelMA + MAO < 5GelMA + MAO < 10GelMA + MAO < MAO. This result indicated that the I_corr_ value of MAO-WE43 alloy was 1.65E-06A, lower than that of MAO-WE43 alloys treated by GelMA hydrogel. Even the best sample was MAO + 1GelMA, which was two orders of magnitude lower than that of MAO-WE43 alloy, which demonstrated that its corrosion resistance had been effectively improved. The E_corr_ of the samples increased in the following consequence: 1GelMA + MAO < 10GelMA + MAO < 15GelMA + MAO < 5GelMA + MAO < MAO. Data showed that the E_corr_ value of MAO-WE43 alloys treated by GelMA hydrogel with different coating layers decreased, in general, compared with that of MAO-WE43 alloy. It was speculated that this might be related to the solvent permeability of GelMA hydrogels. In addition, according to the analysis of corrosion rate: 1GelMA/MAO < 15GelMA/MAO < 5GelMA/MAO < 10GelMA/MAO < MAO, it can be recognized that the corrosion rate of all GelMA/MAO-WE43 alloys was better than that of MAO-WE43 alloy, especially 1GelMA/MAO sample had the lowest corrosion rate. Namely, MAO-coated samples by the GelMA hydrogel treatment remarkably reduced the presence of micropores in MAO coating, blocking the entry of aggressive ions through micropore, and greatly increasing the corrosion resistance of the MAO-coated samples.

### 3.4. In Vitro Biocompatibility

**Figure 9:**
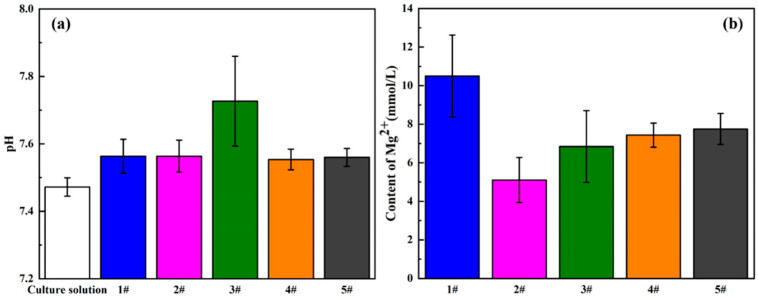
(**a**) pH value and (**b**) Mg^2+^ concentration of the extracts of MAO-WE43 and GelMA/MAO-WE43 samples. 1#: MAO-WE43; 2#: 1GelMA/MAO-WE43; 3#: 5GelMA/MAO-WE43; 4#: 10GelMA/MAO-WE43; 5#: 15GelMA/MAO-WE43.

It is well known that the biocompatibility of magnesium and its alloys is also affected by their degradation behaviors. In the process of corrosion, the rapid changes in pH value and Mg^2+^ concentration will hinder the growth of cells. Herein, the degradation behavior in vitro was evaluated by analyzing the pH value and Mg^2+^ concentration of the extract, as shown in Figure 9. Firstly, the culture medium was presented as the blank, owning a typical physiological pH value of about 7.48, and it could be clearly found that compared with the culture medium, the pH value of the extract increased significantly, especially for the 5GelMA/MAO-WE43 sample, but the other samples exhibited minor differences. As for Mg^2+^ concentration, the result showed that Mg^2+^ concentration of GelMA/MAO-WE43 samples was obviously lower than that of MAO-WE43 samples, and Mg^2+^ concentration of the 1GelMA/MAO-WE43 samples generated by corrosion decreased almost by half with one layer of GelMA hydrogel, which exhibited the sealing effect of GelMA hydrogel coatings. Meanwhile, it can be seen that these results were well in accordance with those obtained from electrochemical tests, further confirming that the GelMA hydrogel coatings had an anticorrosive effect on the MAO-WE43 alloy.

**Figure 10:**
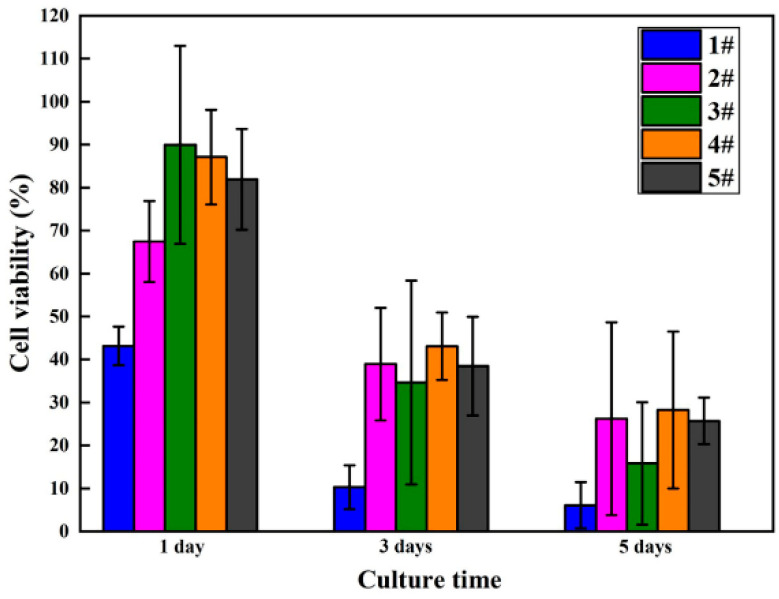
Viability results of MC3T3-E1 cells cultured in MAO-WE43 and GelMA/MAO-WE43 samples for 1, 3, 5 days.

The viability and proliferation of MC3T3-E1 cells cultured in five groups of sample extracts for 1, 3, and 5 days were analyzed employing the CCK-8 assay kit, with statistical results displayed in Figure 10 and Table 4. Compared with MAO-WE43 samples, the MAO-WE43 samples treated by GelMA hydrogel had superior viability and higher cell proliferation. The 5GelMA/MAO-WE43 samples exhibited the highest cell viability, with a value of up to 90% on day 1. Nevertheless, the value of cell viability was 40% and 20% on day 3 and 5, respectively, showing that the cell viability decreased too fast. While, for the 10GelMA/MAO-WE43 samples, relatively high cell viability and a slow decline of cell viability could be obtained. Meanwhile, throughout the entire cell proliferation assay, the GelMA/MAO-WE43 samples showed excellent cell proliferation rate in comparison to the MAO-WE43 samples, especially the 10GelMA/MAO-WE43 samples showed the highest cell proliferation rate on day 5. Therefore, the existence of the GelMA hydrogel layer could provide a similar environment as the cell matrices significantly improved cell viability and proliferation.

## 4. Discussion

### 4.1. Corrosion Resistance

Micro-arc oxidation (MAO) technology can achieve a high degree of combination with the substrate and effectively improve the corrosion resistance of magnesium alloy. Nevertheless, the existence of a porous surface structure limits its long-term corrosion resistance. Therefore, in this study, GelMA/MAO composite coatings are prepared on the surface of MAO-coated magnesium alloy to achieve proper control of its degradation process. Based on the results of morphologies of the samples in Figure 3, Figure 4, Figure 5 and Figure 6, it could be seen that there are micropores and microcracks on the surface of MAO-WE43 coatings, and compared with them, the surface morphology of GelMA/MAO-WE43 alloys with varying layers shows a nonuniform coating with a bit big pores, which is owning to the unfixed GelMA hydrogel viscosity and stress. But overall, the thickness of the hydrogel coating increases with the increase of dip times. The corrosion behaviors of the composite coating are investigated by corrosion measurements. The conclusions are as follows: the results of I_corr_ and corrosion rate show that GelMA/MAO-WE43 alloys improve the corrosion resistance and reduce the corrosion rate, but the E_corr_ result is negatively shifted (Figure 8, Table 3), which may be resulted from the solvent permeability of GelMA hydrogel. According to the electrochemistry theory [37], the E_corr_ of the whole reaction shifts towards the equilibrium potential of the reaction with the larger reaction rate. The present study has found that due to the solvent permeability of GelMA hydrogel water diffusion, the GelMA coating is preferentially attacked in the presence of water. Meanwhile, the hydrogel coatings will absorb, expand, delaminate, and peel off with the diffusion of water. As well known, Mg (OH)_2_ and hydrogen gas will be produced during the degradation process of magnesium alloys, and the overall reaction can be expressed as follows:

Mg → Mg^2+^ + 2e^−^ (2)

2H_2_O + 2e^−^ → 2OH^−^ + H_2_↑ (3)

Mg + 2H_2_O → Mg(OH)_2_ + H_2_↑ (4)

**Figure 11:**
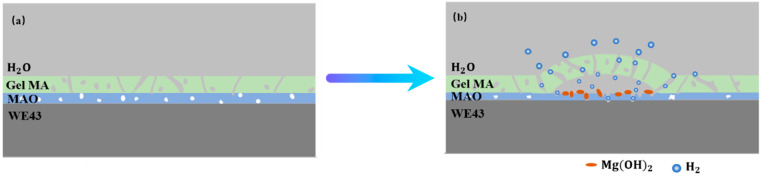
Schematic illustrations of degradation mechanism of the porous GelMA/MAO composite coatings on the WE43 magnesium alloy: (**a**) state of composite coating at the initial stage. (**b**) the blistering and final peeling-off of GelMA hydrogel under the pressure of self-absorption expansion and corrosion products.

The corrosion mainly occurs at the interface of the MAO coating and magnesium alloys, where the corrosion products, such as Mg(OH)_2_, accumulate and can hinder the further corrosion of the MAO-coated alloy to a certain extent, so the corrosion resistance of the magnesium alloy improved. Meanwhile, the H_2_ generated can be expelled through the pores of the hydrogel, while the concentrated H_2_ increases the internal stress under the MAO coatings and GelMA hydrogel coatings if the hole is too small to discharge in time, leading to the generation of expansion bubbles on the GelMA hydrogel coatings. Furthermore, the GelMA/MAO alloys degrade below the GelMA hydrogel layer and exhibit the pitting corrosion characteristics of magnesium alloys, accumulation of products, and swelling of hydrogels, finally leading to destruction and exfoliation. Thus, a degradation mechanism is illustrated in Figure 11, indicating that the degradation of GelMA/MAO-WE43 alloys experiences four steps: (1) water contacts GelMA hydrogel and is absorbed. (2) water diffusing through the micropores of GelMA hydrogel onto the MAO coating and the magnesium alloys. (3) electrochemical corrosion of the MAO coating and magnesium alloys. (4) the swelling and rupture of the GelMA hydrogel.

### 4.2. In Vitro Biocompatibility

An important surface morphology factor related to cell compatibility is roughness. As a general trend, cells are prone to attach and function more actively on rough surfaces [13]. In this study, combined with the experimental results of surface morphology, surface roughness, and cell proliferation, it is found that the surface roughness of GelMA/MAO-WE43 alloys is lower than that of MAO-WE43 alloys. This result is familiar with that of Shang et al. [38], that is, the surface roughness of MAO/GO composite coatings on magnesium alloy surface is smaller than that of the MAO film. However, their cell viability and cell proliferation results are better than those of MAO-WE43 alloys, which indicates that the addition of GelMA hydrogel decreases the surface roughness but promotes the cell proliferation owing to the hydrophilic porous structure. Simultaneously, it is reported that hydrophilic surfaces could increase the surface area of the implants for human osteoblast adhesion. Therefore, as a porous organic material, GelMA hydrogel can not only work as a sealing layer but also provide a great condition for cell adhesion and proliferation on the MAO-coated magnesium alloys. It is well-known that cells are very sensitive to changes in the microsurrounding environment, such as the sharp changes in the pH value and Mg^2+^ concentration. The sharp increase in the pH value and concentration of Mg^2+^ released by the degradation of magnesium alloys and the MAO coatings may hinder cell growth. In this study, the Mg^2+^ concentration of GelMA/MAO-WE43 alloys is lower than that of MAO-coated alloys, and correspondingly, the results of cell viability and cell proliferation have similar rules (Figure 9 and Figure 10). Thereby, as a porous organic material, GelMA hydrogel not only works as a physical barrier layer but also provides a suitable condition for cell adhesion and proliferation on the MAO-WE43 alloys. Combining MAO with GelMA hydrogel can take advantage of the superiority of the two coatings on biodegradable magnesium alloys.

### 4.3. The Balance Mechanism of Corrosion and Coating Protection

It has been supposed that the increase of the thickness of GelMA hydrogel coating would increase the corrosion resistance of the MAO-WE43 alloys, owing to better protection of the coating on the substrates. However, in this study, there is no linear relationship between coating thickness and electrochemical test results, biocompatibility results, etc. For instance, Figure 9 shows that the Mg^2+^ concentration of the MAO-WE43 alloy extract treated by GelMA hydrogel is obviously lower than that of the uncoated samples, and the pH value of the MAO-WE43 alloy extract treated by GelMA hydrogel is higher than that of the uncoated samples. However, the previous studies [29] have proposed that the degradation of MAO/CS coatings gives rise to a decrease in pH value with increasing immersion time and leads to, finally, the MAO/CS coating, keeping the solution’s pH at a medium level. In addition, in this study, with the increase of the coating thickness, the Mg^2+^ concentration gradually increases, but the pH value first increases and then decreases. In general, the 1GelMA/MAO-WE43 alloys have superior corrosion resistance and biocompatibility. That is, the thickness of MAO is not the only factor directly affecting the corrosion resistance and biocompatibility. We speculate that this is due to the balance mechanism between the corrosion of magnesium alloy and the protection of the coating. In detail, GelMA hydrogel can prevent the release of corrosion products more and further protect the MAO-coated alloys with the increase of the thickness of the coating. Nevertheless, as a physical sealing, GelMA hydrogel inevitably generates microcracks during drying; meanwhile, the GelMA hydrogel coatings absorb much water and accelerate the expansion of the coatings, leading to their limited binding force with the substrate, especially for the ones with thicker coating. Finally, it will easily peel-off from the MAO-WE43 alloys. Therefore, it is still challenging to balance the corrosion resistance of magnesium alloys with the long-term protection of GelMA hydrogel coatings by varying the coating thickness in the future.

## 5. Conclusions

GelMA hydrogel coatings with different thickness were prepared on MAO-WE43 alloy substrate by dip-coating method, and the experimental results are as follows:

- The GelMA hydrogel coating plays a role in the sealing of the MAO-coated magnesium alloys, which effectively prevents the entry of corrosive ions, and has more corrosion resistance than the ones without GelMA hydrogel coating.
- GelMA hydrogel coatings can effectively control the Mg^2+^ content of the extract, promoting the cell proliferation and growth, and the good cytocompatibility of the GelMA/MAO-coated magnesium alloys is expected to be a promising bone tissue engineering material.
- There is no linear relationship between coating thickness and biocompatibility results and electrochemical test results consisting of corrosion potential and corrosion rate due to the balance mechanism between corrosion of magnesium alloy and the protection of GelMA hydrogel coatings. Further study may be focused on optimizing the processing of GelMA hydrogel coatings.

## Figures and Tables

**Table 1:** The processing of samples.

Sample	Processing	The Number of Dip-Coating
1#, MAO-WE43	MAO	0
2#, 1GelMA/MAO-WE43	MAO + dip-coating	1
3#, 5GelMA/MAO-WE43	MAO + dip-coating	5
4#, 10GelMA/MAO-WE43	MAO + dip-coating	10
5#, 15GelMA/MAO-WE43	MAO + dip-coating	15

**Table 2:** Average thicknesses of samples.

Sample	The Number of Dip Coating	Thickness (um)
1#	0	13.7
2#	1	14.9
3#	5	15.4
4#	10	16.8
5#	15	25.2

**Table 3:** Parameters of the PDP curves of MAO-WE43 and GelMA/MAO-WE43 samples.

Sample	Processing Mode	Beta A (mV)	Beta C (mV)	I_corr_ (A/cm^2^)	E_corr_ (V)	CorrRate (MPY)	R (omega)
1#	MAO	49.402	148.65	1.65 × 10^−6^	1.5079	1.44470	9.8 × 10^6^
2#	MAO + 1GelMA	191.63	189.42	5.40 × 10^−8^	1.6057	0.047302	7.7 × 10^8^
3#	MAO + 5GelMA	80.638	146.38	3.33 × 10^−7^	1.5437	0.29139	6.8 × 10^7^
4#	MAO + 10GelMA	408.37	337.01	1.60 × 10^−6^	1.5718	1.40000	5.0 × 10^7^
5#	MAO + 15GelMA	110.14	197.61	2.10 × 10^−7^	1.5480	0.18401	1.5 × 10^8^

Beta A: anode slope; Beta C: cathode slope; I_corr_: corrosion current density; E_corr_: corrosion potential; CorrRate: corrosion rate; R: polarization impedance.

**Table 4:** The number of MC3T3-E1 cells cultured in MAO-WE43 and GelMA/MAO-WE43 samples for 1, 3, 5 days (10^4^ cells/mL).

Sample	1 Day		3 Days		5 Days	
	Mean	SD	Mean	SD	Mean	SD
1#	1.647	0.144	1.372	0.551	2.222	1.726
2#	2.430	0.304	4.472	1.418	8.705	7.234
3#	3.155	0.742	4.005	2.570	5.355	4.594
4#	3.063	0.355	4.922	0.850	9.355	5.887
5#	2.897	0.379	4.422	1.242	8.538	1.754
